# Who is saving our streamflow data? Exploring volunteer profiles and their engagement in the SIREN data rescue project

**DOI:** 10.1371/journal.pone.0333091

**Published:** 2025-10-09

**Authors:** Paola Mazzoglio, Miriam Bertola, Tommaso Listo, Luca Princivalle, Luca Lombardo, Alberto Viglione, Pierluigi Claps, Alvise Mattozzi

**Affiliations:** 1 Department of Environment, Land and Infrastructure Engineering, Politecnico di Torino, Torino, Italy; 2 Institute of Hydraulic Engineering and Water Resources Management, Vienna University of Technology, Wien, Austria; Radford University Artis College of Science and Technology, UNITED STATES OF AMERICA

## Abstract

SIREN is a citizen science project that involves lay people in the digitization of historical daily discharge measurements from Italian rivers. Such data, largely available only in printed yearbooks, hinders scientific progress in hydrological studies and water resource management. In this article, we examine the motivations behind citizen engagement in SIREN. Our multi-step approach combines quantitative analysis of online contributions, pilot interviews with selected volunteers, and a comprehensive questionnaire collecting basic demographic data and subjective impressions of the experience. Through these approaches, we identify three participant profiles: two driven primarily by the activity itself and one by the scientific content. The first profile values the straightforward nature of data entry, seeing it as an easy way to contribute with existing skills. The second profile treats participation as a leisure activity, readily fitting into brief intervals of free time. The third profile stems from deeper engagement, encompassing volunteers with professional or personal interests in hydrology, Italian geography, or both. The study also highlights the significant role of retired individuals (an underrepresented group in the citizen science literature) who often contribute using skills developed during their careers. This work highlights the importance of creating citizen science projects that are accessible, meaningful, and connected to volunteers’ lives and interests.

## 1. Introduction

In Italy, since the early 1900s, the National Hydrological and Mareographic Service (SIMN) has been in charge of the management of the Italian monitoring network, whose records were made available in a series of printed hydrological yearbooks. Around 30 years ago, the SIMN was dismantled, and the data collection task was transferred to the regional level [1]. While data collected in recent years is usually available in digital format, historical measurements are often only available in the printed volumes and scanned images of the hydrological yearbooks published by the SIMN. In the past decades, limited efforts have been dedicated to digitize the historical hydrological measurements, even though they would be extremely valuable for supporting water resource management, e.g., by offering insights into past and present hydrological conditions, for the detection of temporal trends in river flows, and for the development and calibration of hydrological models such as the ones used in flood monitoring and forecasting. A few independent digitization initiatives have attempted to recover this information in the past [1–5] making it available in machine-readable formats. Unfortunately, their focus has been limited to specific variables, specific regions and/or targeted years.

The SIREN (Saving Italian hydRological mEasuremeNts) project was thus launched as a concrete attempt to digitize the historical daily discharge data measured along the Italian rivers by leveraging citizen science to crowdsource the digitization of historical daily discharge data. In order to fully understand the potential and limits of such an approach, it is important to examine how citizen science has evolved over time and how it has been applied in hydrological contexts.

The Oxford English Dictionary (O.E.D.) defines citizen science (CS) «*as scientific work undertaken by members of the general public, often in collaboration with or under the direction of professional scientists and scientific institutions*» [6], with the first use of the term (at least with this O.E.D.’s meaning) dating back to 1989 [7]. Such a definition presupposes science as a profession and assumes that professional scientists practice science while the “general public” (or lay people) contributes by assisting with tasks in scientific research. The analysis performed by Kullenberg and Kasperowski [8] records an oscillation in the meaning of the term (that goes from that of science at the service of citizens’ needs and concerns to that of scientific activity participated by a potential and vast audience of non-experts, as well as initiatives more activism-oriented) and a popularity increase in the 2010s, in coincidence with projects using web platforms to reach a vast number of contributors. In this work, we stick to today’s prevailing meaning of participatory activity.

**Fig 1:**
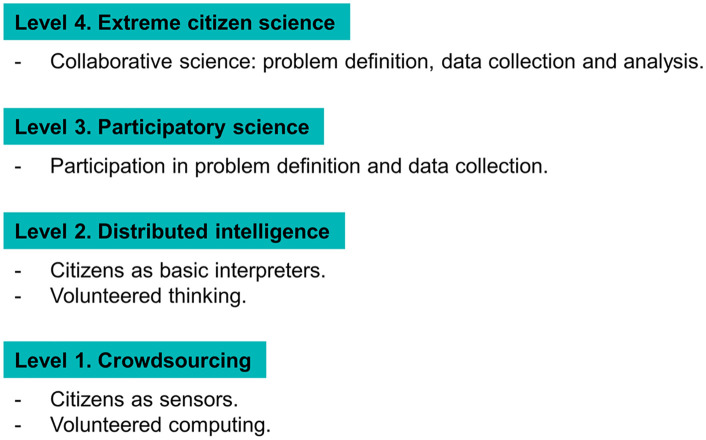
Levels of participation and engagement in CS projects according to Haklay [11].

Over time, diverse classifications of citizen science projects have been proposed, focusing on the kind of participation allowed or on the type of job required. According to Bonney et al. [9], public participation in scientific research can be divided into three main categories that measure an ascending involvement level: contributory projects (usually designed by scientists, for which citizen scientists primarily contribute data); collaborative projects (designed by scientists for which the public contributes with data but also helps in the refinement of the project design, in the analysis of the data, or in the dissemination phase); co-created projects (co-designed by scientists and citizens, with the latter actively involved in the scientific process). Strasser et al. [10], instead, distinguish qualitatively between five different epistemic practices: sensing (to monitor and collect data using sensors), computing (volunteers who share processing powers from their personal computers to make particularly expensive calculations more efficient), analyzing (data analysis work like image classification), self-reporting (sharing of personal data which will then be used in research, especially in the medical field), making (through shared spaces and tools from crowdsourcing/crowdfunding). Haklay [11] proposes an intermediate perspective between the two previous classifications (Fig 1).

In addition to offering a concrete contribution to projects that in terms of time and resources would otherwise be difficult to accomplish [12], CS’s practices are opportunities to disseminate scientific knowledge and awareness to the public. This awareness might not consist just in a public better informed about scientific knowledge, but in performing guidelines for Responsible Research [13], or in questioning clear and fixed separations between the epistemic authority of experts and that of lay people [14], no longer seen as a mere consumer of the scientific activity carried out by professional scientists [10]. This broad vision of CS includes discourses about social reorganization around scientific activity ([15,16]), framings of CS as an alternative to dominant knowledge [17] or as an attempt to bridge the wide gap between democratic deliberations and technical knowledge [18], although the literature seems to overlook or insufficiently explore the most promising forms of participation for this scope, those in which both the interests of participants and the attributions of epistemic authority are redefined throughout the participatory process [19]. Whether or not such an impact could be attributed to CS practices, there is little doubt that work is being done on their institutionalization [13,20,21] and organization, especially through web-portals (see, e.g., [22]).

Within the hydrological field, where examples of structured activities exist (see, e.g., [23]), the typical role of citizen scientists is the collection, the analysis and the interpretation of data on water resources, on weather conditions, or on some component of the water cycle, mainly in terms of quantity and/or quality. These activities include the monitoring of precipitation phase [24], rainfall depths [25], water levels [26,27], water temperature [28], water clarity and color [29], water pH [28] or dissolved hydrogeochemical parameters [28] using simple and low-cost tools such as meters, test kits, smartphones and computers, or collecting water samples for laboratory analyses on nutrient levels, sediment loads, and the presence of contaminants or pollution. In the last decade, several CS initiatives aiming at recovering hydrological and meteorological observations from very old printed and handwritten documents have been launched. Table 1 represents an overview of such initiatives. Most of these projects have been implemented on the Zooniverse platform and involve the transcription of records by the volunteers.

**Table 1:** Citizen science initiatives on hydrological and meteorological data rescue and digitisation.

Project name	Variable(s)	Area	Volunteers involved and period of the activities (as of 30 March 2025)	Link to source and/or reference
Alpine weather news	Meteorological measurements published in local newspaper	City of Bolzano/Bozen, South Tyrol, Italy	408 (March 2023 – April 2023)	https://www.zooniverse.org/projects/yuri-dot-brugnara/alpine-weather-news
AtmosEleC – Atmospheric Electricity for Climate	Long records of atmospheric electric field measurements	Lerwick, Shetland	1158 (May 2024 – ongoing)	https://www.zooniverse.org/projects/hripsi-19/atmoselec-atmospheric-electricity-for-climate
Arctic archives	Weather observations	Greenland	1439 (April 2024 – July 2024)	https://www.zooniverse.org/projects/johandmi/arctic-archives-unraveling-greenlands-weather-history
Climate History Australia	Weather observations	Australia	2263 (September 2020 – July 2021)	https://www.zooniverse.org/projects/caitlinhowlett/climate-history-australia
Digitization Of analogue records In Time (DO IT)	Rainfall, snow and discharge observations	Germany	–	https://doit-lwi.tu-braunschweig.de/en/index.html
Jungle Weather project	Weather observations	Tropical rainforest of the Democratic Republic of the Congo	2506 (April 2020 – May 2020)	https://www.zooniverse.org/projects/khufkens/jungle-weather
Meteororum ad Extremum Terrae (Meteorology of the End of the World)	Weather observations	Argentina	–	https://www.zooniverse.org/projects/acre-ar/meteororum-ad-extremum-terrae; [30]
Old Weather: Arctic	Sea ice and weather observations	Arctic seas	–	https://www.oldweather.org/naval_rendezvous.html
Old Weather: Whaling	Sea ice and weather observations	Arctic seas	–	https://www.oldweather.org/shipping_office.html
Old Weather - WW2	Marine weather observations	Seas and oceans	4463 (December 2020 – December 2021)	https://www.zooniverse.org/projects/krwood/old-weather-ww2
Rainfall rescue	Rainfall observations	UK	17279 (March 2020 – April 2020)	https://www.zooniverse.org/projects/edh/rainfall-rescue; [31, 32]
Southern Weather Discovery	Weather observations	New Zealand, the Southern Ocean and Antarctica	2922 (October 2018 – April 2020)	https://www.zooniverse.org/projects/drewdeepsouth/southern-weather-discovery
Weather Rescue – Oxford	Weather observations	UK	544 (October 2023 – November 2023)	https://www.zooniverse.org/projects/edh/weather-rescue-oxford
Weather rescue at sea	Weather observations	Global	3752 (October 2021 – April 2023)	https://www.zooniverse.org/projects/p-teleti/weather-rescue-at-sea

These diverse applications highlight not only the scientific potential of citizen involvement but also raise important questions about how and why individuals choose to participate. In this context, understanding the nature of engagement becomes essential. Engagement in citizen science is often defined through output measures such as the number of participants, the quantity of data collected, submission rates, web pages accessed, or other appraisals of recruitment, retention, and outreach [33]. However, such a definition is not able to capture the multidimensionality of engagement. In an attempt to provide a simple and clear definition of this term, [33] suggested a definition of engagement in citizen science as the “*emotional, behavioral, cognitive, and social experiences that initiate and sustain lifelong learning and that are largely influenced by motivational factors*”. Thus, engagement is strictly linked with the motivations, but the motivations themselves can not guarantee continuous engagement.

In recent years, few research works addressed the investigation of the motivations that lead people to be engaged in citizen science projects. [34] highlighted, in a review article based on peer-reviewed publications on environmental monitoring in developed countries published in the year 2005 or later, that intrinsic motivation (meaning that an activity is undertaken out of interest, enjoyment, or satisfaction) is the strongest one. [33] pointed out that this is particularly true in contributory and collaborative CS projects. [35], however, found that intrinsic motivation mainly influences the quantity of participation, but that it did not affect the quality of participation (i.e., the accuracy and precision of contributions). [36] pointed out that the scientific activities offered in a CS project are often only sufficiently attractive for some participants: to improve the intrinsic motivation for participation and at the same time enhance participants’ learning, project administrators must offer multiple engaging learning opportunities that can address the needs of all target groups. Personal interest in the topic or in the particular project also motivates people to be involved, together with recognition and attribution of their contributions [34,37–39]. In the same review paper, [34] recognized that several other extrinsic motivations emerged from the interviews carried out within several citizen science projects, such as learning, knowledge exchange, the urge to make a contribution (engagement provides individuals with a sense of purpose and contribution to a larger cause) or ego-involvement (volunteers can have a feeling that they are needed or that their friends will think positively about them if they participate in these activities). [40] pointed out from the experience gained during a case study that the motivations behind citizen scientist involvement depend on the country: citizen science in developed countries is a pastime, while citizen observers in developing countries very often earn an income from their work.

In the literature, an in-depth understanding of the motivations behind individual participation in initiatives like SIREN is still missing. Why do people choose to contribute with their time and effort to digitizing historical data? What drives their engagement in a citizen science project in the field of environmental sciences? Can we identify common profiles when analysing the different contributors?

To address these questions, in this work we conducted an anonymous survey among SIREN project participants. This survey was designed to reconstruct contributing practices, and, from there, to capture a comprehensive view of the factors influencing contributors’ involvement, including personal interests, perceived benefits, and the overall appeal of the project and, more in general, of the citizen science initiatives. By analyzing survey responses, we aim to understand the key motivations that drive citizen scientists to engage with the SIREN project. Our goal is to provide actionable insights that can enhance the design and effectiveness of similar citizen science initiatives.

## 2. Methodology

**Fig 2:**
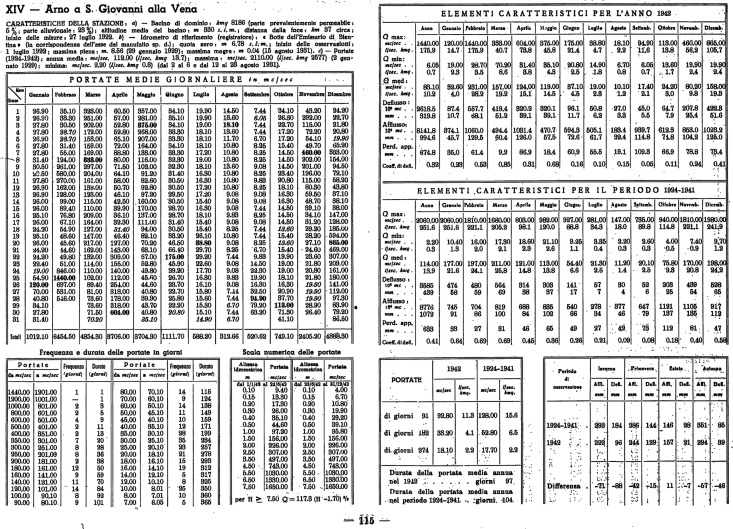
Example of a page of the Italian hydrological yearbooks containing data acquired during 1942 by the gauging station named “Arno a S. Giovanni alla Vena”. Although the pages of the yearbooks may have different structures, they typically report the gauging station name at the top of the page and the table of the daily discharge measurements (“Portate medie giornaliere” in Italian) for one year just after the station name. Other information, e.g., flow statistics or characteristics of the station may be present (e.g., the tables titled “Frequenza e durata delle portate in giorni / Frequency and duration (in days) of the discharges”, “Elementi caratteristici per l’anno 1942 / Statistics of the year 1942”, “Elementi caratteristici per il periodo 1924-1941 / Statistics of the period 1924-1941”, etc.).

As mentioned in the Introduction, the SIREN project aims to digitize the historical daily discharge time series from the Italian historical hydrological yearbooks and to produce a consistent and open-access dataset. The yearbooks contain more than 17.000 tables with daily discharge data (each table typically contains the measurements collected in one year by one gauging station). Fig 2 shows an example of a typical yearbook page containing discharge measurements. From this project, we expect to retrieve observations of about 1000 stations installed along the Italian rivers between the 1920s and 1990s.

After a review of all the freely available tools that can assist this type of project, we selected the user-friendly Zooniverse platform. The reasons that led us to choose this platform include its suitability for digitization projects like SIREN, without having the necessity of developing custom tools, e.g., to automatically assign pages to different volunteers. Additionally, the Zooniverse community is highly active, and the anonymous review process conducted by a small group of volunteers before the official launch (a mandatory step needed to be listed as an official Zooniverse project) allows researchers to test the workflow with individuals of various ages, nationalities, backgrounds, and experiences. We also chose the Zooniverse platform for its strong commitment to user privacy. Participants can contribute either by creating an account or anonymously, without the need to log in. For registered users, the only information visible to the project team is the chosen username and the timestamp of each digitization session. No additional personal data (such as email addresses, location, or demographic details) is shared with project managers. This privacy-by-design approach aligns well with the ethical standards of our research and ensures that participants can engage with the project while maintaining full control over their personal information.

To make the yearbooks easily accessible to the volunteers, we uploaded a scanned version of all the tables on the Zooniverse project page. The platform randomly assigns a page to the volunteer who enters the digitization workflow, and the digitization work starts. The task that we designed is quite simple: in the first step, we ask volunteers to identify the station name (usually reported in the top left corner of the page) and, in the following steps, we ask them to digitize one month of daily discharge data. Each observation is digitized by at least two different volunteers, who can also insert flags for uncertain or unreadable values and report problems encountered during the digitization. All the inconsistencies among redundant transcriptions, as well as reported flags and problems are manually checked by the project team to ensure that the data have been properly digitized. For these reasons, SIREN can be considered a “level 2” citizen science project [11, Fig 1] based on volunteered thinking and, in certain cases, also interpretation.

**Fig 3:**
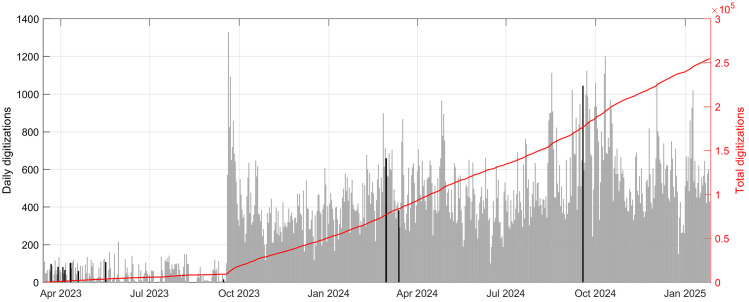
Number of daily contributions to the SIREN project. In black, the days in which we advertised the project on social media or on blogs of the sector. The first beta version was launched on 22nd March 2023, in the occasion of the 2023 World Water Day. The final version was released on 19th September 2023.

Fig 3 shows the daily contributions (in terms of number of months digitized) with the indication of the main advertising activities in black color. The project was advertised in scientific conferences, media channels and blogs of hydrological sciences. From Fig 3 we can not see an increase in the digitizations following the advertisements. We hypothesized that people from Academia, who were mainly reached by these dissemination activities, rarely have the time or interest to contribute as anonymous volunteers to other research projects. On the contrary, we noticed a marked increase in the number of digitization starting from the day of the official launch on the Zooniverse platform in late September 2023, confirming the value of the Zooniverse community. From Fig 3 we can also see a modest decrease in the number of daily digitization after one week from the launch, probably due to new projects launched on the platform that divert the attention of the users. Thus, we decided to investigate the motivations that drive volunteers to be constantly involved in these initiatives.

Our analysis of engagement in the SIREN project employed a multi-step methodology: i) data analysis; ii) pilot interviews; iii) an anonymous survey.

In the first step, we approached the investigation with a quantitative data analysis: we collected and analyzed the digitization data from the Zooniverse platform to identify the contributions of each registered anonymous volunteer (with this term we identify the contribution performed by volunteers with a Zooniverse account; the term anonymous is used to highlight that for those users we only know the username).

Based on the findings of the data analysis, we conducted a few pilot interviews with selected individuals identified from the contribution’s statistics. The selection of subjects for interviews (whose only known information was their username on Zooniverse) was guided by the criterion of including at least one intensive user and a couple of users with a lower number of entries. Recruitment for the interviews lasted from 23^rd^ December 2023–15^th^ January 2024. Recruitment was carried out by contacting SIREN participants via the Zooniverse platform, where users are present anonymously. Three people agreed to be interviewed. The three participants gave their consent to the processing of personal data, under the guarantee of the European GDPR, by signing an informed consent document.

We scheduled an hour-long interview with each of them to gain deeper insights into participant motivations and experiences. We chose the method of semi-structured interviews [41], presenting ourselves to the interviewees as social scientists trying to understand how to gain a better understanding of how CS projects function, with the goal of enhancing the user experience of activities like those on Zooniverse – which was also a point of the research.

The interviews were structured around three main narrative blocks [42]. The first block included questions about their experience with data entry for the SIREN project, their interaction with the web interface, the consistency of these operations over time, and the amount of time they dedicated to this activity. The second block focused on citizen science projects in general, asking whether they had participated in other projects (either on Zooniverse or elsewhere) and if those were similar to SIREN. The final block of questions delved into the respondents’ interests in participating in SIREN and similar initiatives, whether their interest stemmed from the specific topic, environmental issues, or other factors.

The interviews have been mainly analyzed through grounded theory [43].

Based on the findings of the previous step, we structured an anonymous questionnaire (S1 File) around two hypotheses: 1) that a significant portion of SIREN contributors shared aspects of this profile, and 2) that there was another segment with different profiles that we had not yet identified and needed to uncover.

The survey was launched on the 20^th^ of March 2024 and closed on the 10^th^ of April: a banner that advertised it was inserted in the Zooniverse platform. To incentivize participation, 284 Zooniverse users among the most active were contacted via private message (277 out of 284 digitized more than 10 months of data), asking them to fill out the form in their spare time. The questionnaire collected information anonymously, an aspect explained to participants in a recruiting message on Zooniverse’s SIREN project page.

## 3. Results

### 3.1 Data analysis

**Fig 4:**
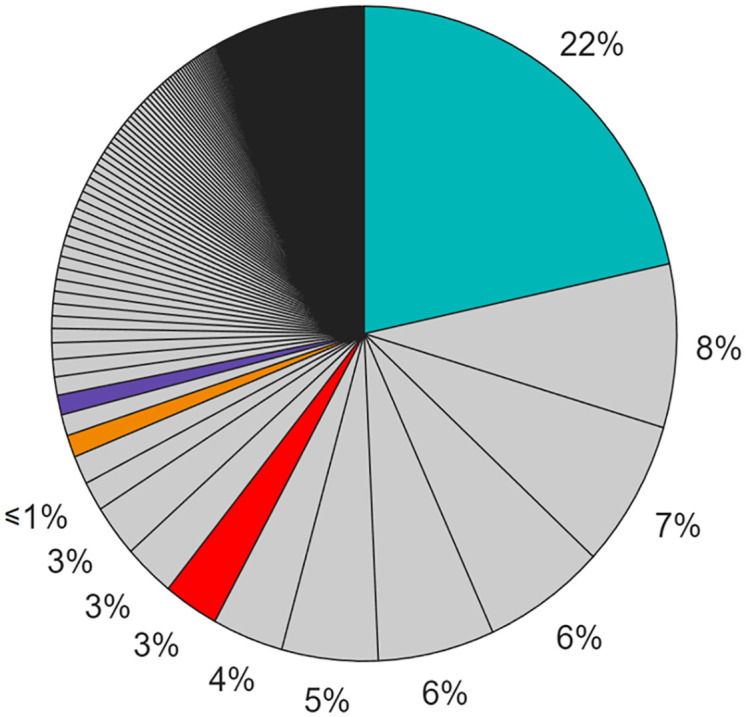
Contributions of each volunteer, expressed in percentage. In red, the 3% of the contributions that were made by users who did not log in. In light blue, orange, and purple, the contributions of three selected volunteers further analyzed in Fig 5.

**Fig 5:**
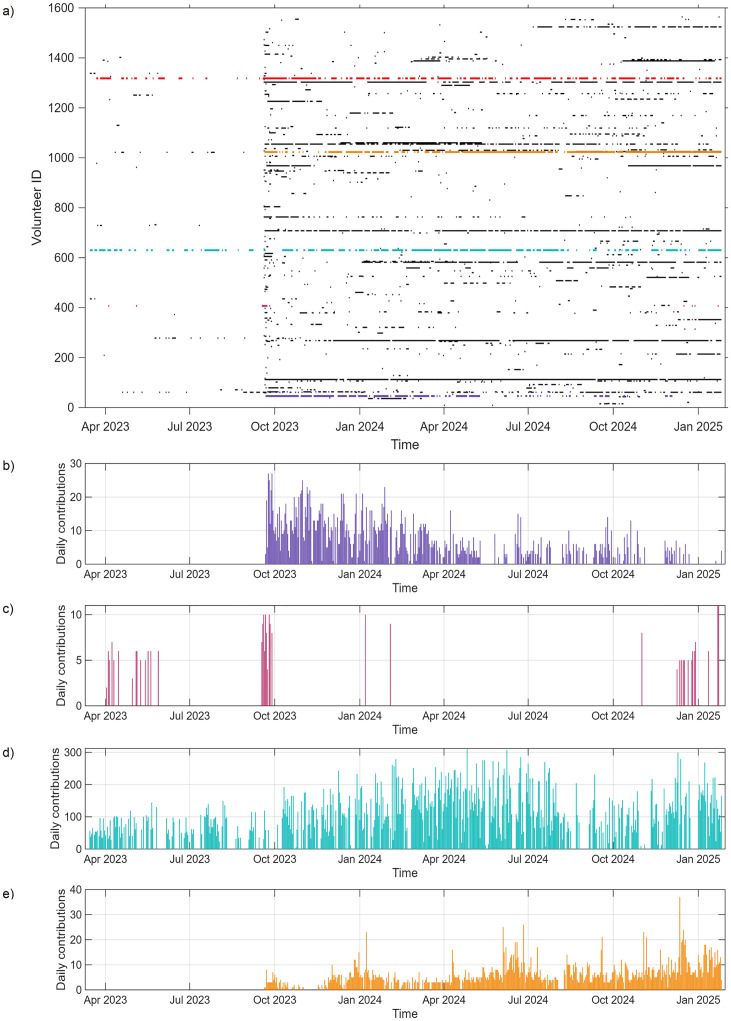
Temporal patterns of user activities in the SIREN project. Panel a shows daily engagement across all volunteers (each horizontal line represents one volunteer, and each vertical column corresponds to a day in the project timeline). Cells are colored black when the corresponding volunteer contributed to at least one digitization task on that day, and white when no contribution was made. Contributions made by non-logged-in users are indicated in red, distinguishing anonymous inputs from those traceable to registered accounts. Panels b–e present the daily number of digitized months for four selected volunteers, illustrating different patterns of contribution intensity and consistency.

As of the 26^th^ of January 2025, 1570 unique registered volunteers contributed to the digitization of at least one month of data. Users who did not log in before entering the digitization workflow can not be tracked. It is interesting to notice that the 6 most active users digitized about 50% of the data, and the 100 most active users digitized about 90% of the data (Fig 4). If we focus on the lower parts of the ranking, we notice that 729 registered users digitized 3 or fewer months. These data suggest that most of the work is performed by few loyal volunteers. The temporal evolution of the digitizations is visible in Fig 5. Fig 5a highlights heterogeneity in user activity, ranging from short-term engagement to sustained participation over long periods. Fig 5b–5e show instead individual time series and allow for a closer inspection of diverse engagement trajectories, including regular versus sporadic participation.

### 3.2 Pilot interviews

From the transcriptions of the interviews, we highlighted thematic cores and noted some interesting and unexpected patterns. First, the profiles of the three interviewees showed commonalities: they were women, retired, and had jobs related to data entry. It is worth remembering that the Zooniverse profiles from which they were selected are anonymous. Second, none of the three expressed particular interest in hydrology as a subject. As we explored topics that might align more closely with their interests, we noticed a recurring theme: rather than the subject matter itself, it was the practical tasks required by SIREN, such as copying and data entry, that contributed to the enjoyment they derived from participating in the project. All three interviewees found rewarding in their roles as contributors that they were offering concrete value to the project, as SIREN activities required skills they had developed in their professional lives.

We found these insights relevant and worthy of further investigation. However, we also acknowledged the possibility that these profiles, while similar to one another, might not be fully representative of broader participation in SIREN.

### 3.3 Anonymous survey

After a few weeks from the survey publication, we received 116 responses, comprising 49 partial and 67 complete responses. Some partial responses were deemed potentially useful for a first investigation since they replied to most of the questions and stopped answering only in the last phases, while all the others were discarded since the volunteer stopped answering after a few initial questions. In total, we obtained 69 useful responses (67 complete and 2 incomplete, see S2 File).

The gender identity of the volunteers was: 38 out of 69 (55%) female, 24 out of 69 (35%) male, 2 out of 69 (3%) non-binary, while 5 preferred not to answer. The female component is thus clearly predominant.

**Fig 6:**
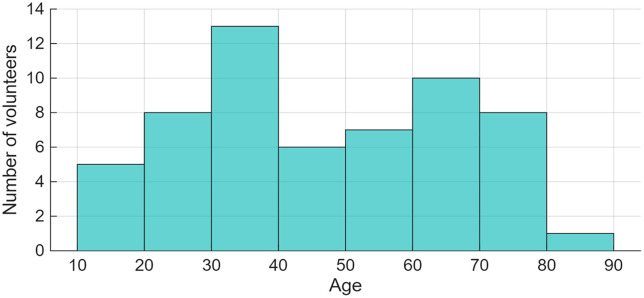
Age distribution of the volunteers.

The dataset includes 58 valid responses to the question related to the age, with missing values and entries coded as −1 excluded from the analysis. The average age of respondents is 46.7 years, with a median age of 45.5 years and a standard deviation of 19.4 years. The youngest participant is 16 years old, while the oldest is 81. Overall, the sample covers a broad age range, encompassing almost the entire adult life course. The age distribution is visible in Fig 6.

If we focus on the employment status:

- 29 out of 69 (42%) are employed full-time or part-time or self-employed;
- 19 out of 69 (28%) are retired;
- 8 out of 69 (12%) are students;
- 1 out of 69 is unemployed;
- 6 out of 69 (9%) fall in other categories;
- 6 out of 69 (9%) preferred not to answer.

The majority of the volunteers are thus employed or students, while about a quarter are retired.

Almost all the volunteers usually work on SIREN from their home (62 out of 69, i.e., 90%) or from work (18 out of 69, i.e., 26%), while only a few of them work from public spaces (like libraries, cafes, or other recreational spaces) or from public means of transportation. Here, the total exceeds 100% because respondents were allowed to select more than one location, indicating that some volunteers contribute from multiple places depending on the context or availability. 36 out of 69 (52%) of them usually do other activities on the side or in the background when working on SIREN, like watching TV, listening to music, or talking with someone. 5 out of 69 (7%) of them found the activities requested during the digitization workflow akin to activities that they are/were used to perform for their job, while 26 out of 69 (38%) of them found a partial similarity.

Participants were drawn to the SIREN project for a variety of reasons, reflecting a range of personal interests and professional backgrounds. Here are the main points of interest that motivated their participation:

- interest in data digitization and climate studies: many participants were attracted to the project due to their interest or expertise in digitizing data, while others found it interesting since in the past they were already involved in climate and hydrological data digitization. They appreciated the straightforward, easy-to-understand tasks with clear instructions, which allowed them to contribute meaningfully to scientific research;
- connection to personal and professional backgrounds: some contributors had backgrounds in environmental science, hydrology, or related fields. These participants were motivated by the relevance of the project to their professional interests and research areas. Others were drawn by past experiences with similar projects, such as digitizing weather data or contributing to environmental data projects;
- citizen science and volunteering: the value of citizen science and the opportunity to contribute to important scientific research were significant motivators. Participants enjoyed the feeling of making a positive impact, even in a small way, on understanding climate change and preserving environmental data;
- personal interests and hobbies: some contributors were motivated by their personal interests, such as a fascination with weather data, natural disasters, or Italian geography. The project offered a way to engage with these interests while contributing to scientific research;
- ease and simplicity of the task: the simplicity and accessibility of the tasks were appealing to many. Participants liked that the tasks were easy to complete, required minimal effort, and could be done in short bursts of free time. This made the project a convenient and enjoyable way to contribute;
- invitation and word of mouth: a few participants joined the project after being invited by family members or learning about it from professors or online platforms like Zooniverse. This highlights the importance of personal networks and community engagement in attracting volunteers;
- nostalgia and personal connection: for some, the project evoked nostalgia for past experiences or personal connections to environmental studies. This emotional connection provided additional motivation to contribute.

Most users do not seem overly interested in counting their entries, suggesting that gamification factors are unlikely to be a significant motivation.

We asked participants how they felt after leaving the SIREN digitization workflow, and we received a range of different responses. The majority of them expressed positive feelings, such as accomplished, satisfied, relaxed, fulfilled, content, and glad. A few noted feelings of fatigue and exhaustion. Some participants expressed mixed emotions, combining satisfaction with other feelings such as curiosity, competitiveness, or frustration. Other notable responses included those who stopped due to external reasons like family time or physical discomfort. A small number of respondents felt neutral or indifferent. When answering this question, respondents were not constrained by predefined options and could freely express their thoughts in their own words. This unrestricted format allowed for diverse, personal responses. It is particularly noteworthy that, despite this freedom, many participants provided similar answers, often describing feelings of accomplishment, satisfaction, or fulfillment. This consistency across responses suggests a shared positive experience among contributors and highlights the intrinsic rewards associated with participating in the SIREN project.

**Fig 7:**
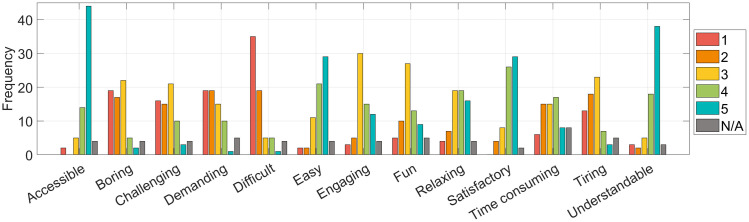
Answers to the question “How much do these terms apply to SIREN? Please rate, on a scale from 1 to 5, where 1 means that the term does not apply and 5 means that applies a lot, the following terms.”.

Participants were asked to assess how much a series of terms applied to their experience with the SIREN project (Fig 7), using a 5-point Likert scale from 1 (the term does not apply) to 5 (the term applies very much). Overall, the most strongly associated terms were “accessible” (mean = 4.51), “understandable” (4.30), “satisfactory” (4.19), and “easy” (4.12), indicating that volunteers generally found the platform clear, usable, and rewarding. Conversely, the lowest scores were recorded for “difficult” (1.74), “demanding” (2.30), and “boring” (2.29), suggesting that the task was not perceived as particularly fatiguing or cognitively heavy. Intermediate scores were assigned to terms like “engaging” (3.43), “fun” (3.17), and “relaxing” (3.55), indicating that while the activity was generally positive and pleasant, it was not necessarily considered exciting or entertaining by all.

The volunteers acknowledged the use of various tricks to enhance efficiency during the digitization process. It is interesting to notice that most of them dedicated time to detail the tricks they are using, probably in an attempt to provide project administrators with potentially useful information that can be shared with the community, or because they are proud of having identified them. A common approach was using copy-and-paste for repeated numbers to save time and reduce errors. Many are used to zooming in on the image, aligning the month to the screen’s edge, or showing only 10 numbers at a time to maintain focus and accuracy. Some used optical character recognition (OCR) tools, although this often required significant corrections. Visual aids like holding half sheets of paper over the screen or dividing the image into sections with lines drawn in software like Microsoft Paint were also mentioned to avoid mistakes and keep track of progress. Others verified their entries by counting and comparing blocks of ten numbers, ensuring no entries were missing or duplicated.

Participants also employed various tools to enhance their speed and accuracy during the digitization process. A significant number relied on the number pad for quicker numerical entry, finding it faster and more convenient than the horizontal number keys above the letters on the keyboard. This method allowed them to focus on the screen without needing to look down at the keyboard. Some participants used dual screens or split their screen to manage the data entry process better. Additional tools included the mouse for navigating and zooming in and out, as well as pointing with fingers or a pencil to keep track of their position in the data.

Participants in the SIREN project reported a variety of learning outcomes. Many individuals improved their familiarity with Italian geography, including the names of months, places, and rivers. Some participants, particularly those learning Italian, found the project beneficial for language practice. Several contributors became more efficient with data entry techniques, learning to utilize tools like copy-paste functions and the number pad for faster input. They also gained insight into the methods of digitizing historical data and the importance of such efforts for scientific research. Participants also appreciated the historical aspect of the data, noting the meticulous records kept and the variability in river flows. They expressed admiration for the dedication of past scientists and the extensive data collection on even small rivers. The project also highlighted the differences in river dynamics and flow rates across various locations and times, which was something volunteers were not aware of.

Through the analysis of the questionnaires, we were able to identify three distinct participant profiles. Two of them are mainly activity-related, while the third is content-related.

The first one is composed of individuals who are not so much interested in the research topic, but are willing to contribute to a project considered useful through their skills without too much effort. Sentences like *“It was a simple task of transcribing numbers that I could do with little effort*” and *“I was looking for a transcription project on Zooniverse and SIREN looked interesting*” were common in this group. This group consists also of former data-entry employees (“*As several of my Zooniverse project contributions, I found I was drawn by the act of data entry. Many years ago, I was a data entry operator and perhaps that is what grabbed my attention. I also appreciate that I don’t have to draw conclusions - just transfer the information!*”). This group values the straightforward nature of the work and the chance to remain involved without significant cognitive load.

The second one consists of those who use it as a leisure activity in their daily lives, especially during spare time or breaks in the working hours, recognising the ease of use and speed with which they can contribute (one respondent noted that SIREN is “e*asy to use, useful, a good break*”, while another one said “*I work Zooniverse projects during lulls at work. I look for projects that can be completed in short bursts, that provide clear instructions, and that can be picked up at intervals without losing the thread of what I was doing*”).

The third is made by those who are professionally interested and contribute on the basis of an understanding of the research topics and objectives, underscoring a deeper engagement with the scientific objectives (“*I’m interested in hydrological data*”, “*I study hydrology and am learning Italian so it seemed pretty cool and similar to my situation currently!*”). In this case, factors in professional solidarity also emerge. In this third profile, we also included a few people who feel a relation to Italy (“*I’m often volunteering for many causes on Zooniverse, but SIREN is the first one that is strictly related to Italy (my country) so I hope to have been useful in helping control river conditions*”).

Statistical methodologies were used to analyse the results of the questionnaire to explore the relationship between sociodemographic characteristics and a set of subjective indicators related to the participants’ experiences in the project.

The focus was placed on the three independent variables (gender, age, and employment condition), and only the 58 answers with complete information regarding the independent variables were considered. Within this subset, the gender distribution is: 34 out of 58 (59%) female, 22 out of 58 (38%) male and 2 out of 58 (3%) non-binary. As for the complete sample described at the beginning of this section, the average age of respondents is 46.7 years, with a median age of 45.5 years and a standard deviation of 19.4 years. The youngest participant is 16 years old, while the oldest is 81. If we focus on the employment status:

- 27 out of 58 (47%) are employed full-time or part-time or self-employed;
- 17 out of 58 (29%) are retired;
- 7 out of 58 (12%) are students;
- 1 out of 58 is unemployed;
- 6 out of 58 (10%) fall in other categories.

The three independent variables were tested against 13 dependent variables measuring participants’ subjective experience with the project (e.g., “accessible”, “boring”, “challenging”, “demanding”, “difficult”, “easy”, “engaging”, “fun”, “relaxing”, “satisfactory”, “time consuming”, “tiring”, “understandable”, as shown in Fig 7) evaluated on a 5-point Likert scale. In addition to analyzing the continuous responses, a binary (dummy) version of each variable was also created. This resulted in a total of 26 dependent variables (13 continuous and 13 binary) used in the analysis.

To explore potential differences in user perceptions based on demographic characteristics, we conducted a series of independent samples t-tests comparing male and female respondents. Specifically, we tested whether gender was associated with differences in how volunteers rated various aspects of the digitization activity (e.g., whether it was perceived as “engaging,” “relaxing,” “challenging,” etc.). For each variable, we compared the mean responses between the two groups and computed the t-statistic and corresponding p-value (Table 2).

**Table 2:** Results of the t-test analysis performed to investigate user perception (evaluated on a 5-point Likert scale) based on demographic characteristics.

Variable	Mean value of the women sample	Mean value of the male sample	p-value	Test passed
Accessible (Likert scale)	4.72	4.23	0.0466	Yes
Boring (Likert scale)	2.00	2.43	0.1628	No
Challenging (Likert scale)	2.27	2.57	0.3585	No
Demanding (Likert scale)	2.15	2.30	0.6518	No
Difficult (Likert scale)	1.48	1.95	0.1218	No
Easy (Likert scale)	4.45	3.71	0.0162	Yes
Engaging (Likert scale)	3.79	3.15	0.0267	Yes
Fun (Likert scale)	3.39	3.05	0.2743	No
Relaxing (Likert scale)	3.94	3.15	0.0146	Yes
Satisfactory (Likert scale)	4.36	3.86	0.0539	No
Time-consuming (Likert scale)	2.97	3.26	0.4095	No
Tiring (Likert scale)	2.06	2.75	0.0197	Yes
Understandable (Likert scale)	4.24	4.41	0.5275	No

From Table 2 emerges that women evaluated the experience as more relaxing, engaging, easy and accessible, while men found it more tiring. No significant differences were found for the other variables. Results obtained with the binary (dummy) version of each variable are not reported since they do not provide relevant additional information.

To explore potential associations between age and the subjective experience of volunteers, we computed Pearson correlation coefficients between the age of the respondents and each of the variables describing their experience with the digitization task (Table 3). Before performing the correlation analysis, missing values were removed.

**Table 3:** Results of correlation analysis between age and user perception. Only the applications that resulted to be statistically significant are here reported.

Variable	Correlation	p-value	Test passed
Challenging (binary scale)	−0.357	0.007	Yes
Difficult (binary scale)	−0.293	0.0286	Yes

From Table 3 emerges that as age increases, participants tend to perceive the experience as less challenging and less difficult.

To investigate whether subjective experiences differed across employment categories, we performed a one-way Analysis of Variance (ANOVA) for each of the dependent variables. Prior to conducting the ANOVA, we excluded any groups with fewer than 5 valid responses to ensure statistical robustness. For each variable, an ANOVA model was fitted using employment status as the independent factor. When the ANOVA yielded a statistically significant result (p < 0.05), a Tukey HSD test was applied to identify which specific employment categories differed significantly from one another. Overall, none of the ANOVA tests produced statistically significant results at a 5% level. In most cases, the p-values were well above this threshold, indicating no significant differences between groups.

Although some statistically significant differences emerged from the analysis, it is important to interpret these results with caution. The relatively small sample size limits the generalizability and statistical power of the findings, particularly in detecting complex relationships among variables. While the analyses provide valuable preliminary insights, they should be considered exploratory rather than definitive. Future research involving larger and more diverse samples would be essential to validate these initial observations, strengthen the reliability of the results, and allow for more nuanced statistical modeling. Such studies could also explore additional factors that may influence participants’ engagement and experiences in citizen science projects.

## 4. Discussion

These profiles confirm what has already been observed in [13], namely that citizen science is linked to broader social trends such as the expansion of education, increased leisure time, attention to personal well-being, and, above all, widespread access to digital technologies and web applications. These developments have opened new avenues for participation in scientific research by enabling individuals to contribute from home or other informal settings. However, it is important to note that these opportunities are not equally accessible to the entire population, but rather to more economically affluent social groups (this, in itself, serves as a criterion for access to CS activities, which has not been explored here, although it may be partially inferred from certain data collected through the questionnaire).

This study also revealed the significant presence of what we defined as an activity-related profile and, in part, highlights a potentially original aspect of it in comparison to the reviewed literature. More specifically, in terms of activities, we observed that participant engagement was motivated not so much by the opportunity to learn new skills as an investment for the future, but rather by the desire to refine and enhance skills and competencies learned in one’s professional life.

Related to motivations, participation is often analyzed as a form of investment in the future, as participants gain new skills, knowledge, or insights through their involvement. In contrast, for the group of individuals who engage in this project to leverage their existing expertise, SIREN is viewed as an investment in the present. Rather than focusing on learning something new, their motivation lies in applying and refining the skills they already possess, deriving satisfaction from contributing to a meaningful initiative in real-time. This distinction highlights the appeal of SIREN for participants who seek immediate purpose and productivity, rather than long-term personal development. By offering opportunities to utilize pre-existing competencies, the project serves as a platform for participants to channel their expertise into impactful scientific work, enabling also participants to remain professionally and intellectually active.

Furthermore, this work sheds light on a demographic that is often overlooked in the design of CS initiatives: retirees. A review of the literature reveals limited evidence that retirees are significantly engaged in citizen science activities, particularly online. Indeed, most of the CS projects consider youth as one of the main targets [44–46]. However, this project highlights a notable exception, as a substantial proportion of contributors are retired individuals, many of whom bring with them decades of work experience in relevant fields. This finding suggests that online citizen science projects, such as SIREN, may hold appeal for retirees, offering them opportunities to apply their skills, stay mentally active, and contribute to scientific initiatives. This piece of evidence underscores the importance of considering retirees as a valuable and underutilized resource in the design and promotion of future citizen science projects.

In sum, within this work, the responses have highlighted some previously unseen participant profiles and patterns of engagement, which can inform future iterations of the project and similar citizen science initiatives. These findings serve as a new hypothesis and starting point for exploring the diversity of motivations and experiences among contributors. Despite these new insights provide a new point of view for understanding the motivations and characteristics of participants in the SIREN project, the scope of the analysis is limited to some qualitative insights by the relatively small sample size of 69 valid responses, which is insufficient for conducting in-depth statistical analyses since it lacks the statistical power needed to draw robust conclusions or identify broader trends with high confidence. Future work could focus on expanding the sample size to strengthen the reliability of the findings and allow for a more comprehensive statistical evaluation.

Despite the limitations, this analysis represents an important step toward understanding the complex and multifaceted dynamics of volunteer engagement in citizen science projects. It underscores the importance of designing projects that not only welcome broad participation, but also recognize and support the diverse ways in which different people (depending on their background, experience, and life stage) may wish to contribute to science. Further research building upon these initial observations will be critical for deepening our understanding of how citizen science can inclusively engage and sustain a wide range of participants.

## 5. Conclusions

In this work we investigated the motivations behind citizen engagement and the possible presence of volunteer profiles in the SIREN project, a citizen science initiative that aims at digitizing the historical daily discharge measurements collected along the Italian rivers, available only in printed format, thus not easily accessible by researchers, practitioners, and policymakers.

The investigation consists of a multi-step methodology: i) analysis of quantitative data on citizen contributions; ii) three pilot interviews with selected individuals identified from the contributions statistics obtained in the previous step; iii) a comprehensive questionnaire open to a broader audience to verify and expand upon the insights gained from the pilot interviews.

Overall, the SIREN project attracted a diverse group of participants, each bringing their own unique motivations and interests to address the same common objective. Through the analysis of the questionnaires, we identified three distinct participant profiles in SIREN, two primarily activity-related and one content-related. The first group leverages its data entry skills with minimal effort and shows limited interest in the research topic, while the second views digitization as a leisure activity easily integrated into short breaks. The third profile demonstrates deeper engagement, with volunteers who possess professional or personal interests in hydrology or Italian geography. Many participants regard SIREN as an investment in the present, focusing on applying existing skills rather than acquiring new ones.

Contrary to literature suggesting limited retiree engagement in online citizen science, a notable proportion of SIREN’s contributors are retired individuals, highlighting their potential as an underutilized resource.

To further investigate relationships between demographic characteristics and subjective experiences, we conducted statistical analyses on survey responses. T-tests, correlation analyses, and ANOVA tests were applied to assess the influence of gender, age, and employment status on users’ perception of the digitization activity. While some statistically significant results emerged (such as women reporting the experience as more relaxing and engaging, and older users finding it less challenging) the overall explanatory power of demographic factors was limited. Nevertheless, these analyses provided useful quantitative support to the qualitative insights, reinforcing the importance of considering both perspectives when designing inclusive citizen science projects. The results presented in this work highlight the importance of creating citizen science projects that are not only scientifically meaningful but also tailored to the needs, interests, and capacities of diverse participant profiles.

To conclude, we would like to emphasize that exploring the relevance of the activity-related profile, as it has emerged in this context, holds particular promise for future projects. This approach could offer a fresh perspective on organizing participatory activities between scientific research and society at large. It goes beyond the traditional, linear model of citizen science, where expertise is confined to scientific institutions. Instead, it diversifies and enriches the process by highlighting specific activities, thereby incorporating expert contributions in both directions.

## Supporting information

S1 File The file contains the questionnaire that we shared with the citizen scientists. (PDF)

S2 File The file contains the answers to the questionnaire. (XLSX)
