# Supplementary material for: Who is saving our streamflow data? Exploring volunteer profiles and their engagement in the SIREN data rescue project
Source: PLoS One. 2025 Oct 9;20(10):e0333091. doi: 10.1371/journal.pone.0333091 (PMC12510526; doi:10.1371/journal.pone.0333091)
Supplement: S1 File — (PDF) [file pone.0333091.s001.pdf]

Introduction

We ask you to try to recall your last session on SIREN. Please take between 30 seconds and 2 minutes to rethink about the last time you contributed to SIREN. When you have done it and feel ready, click the rectangle below and start.

1/4 Your last session on SIREN

G02Q01

Among the following actions, which ones did you perform the last time you were on SIREN? Please choose those actions that you performed from the column on the left and arrange them in the column on the right, from top to bottom. On the top, you will have your first actions, and on the bottom your last (you don't need to order them all, but only the ones you actually do).

- login
- looking around Zooniverse
- looking at the "About" section
- looking at statistics regarding SIREN's project completion
- looking at personal statistics
- looking at SIREN's instructions ("Field Guide", "Tutorial")
- going to "Classify"
- open a page/image to digitize
- digitizing /images inserting stations' names and discharges' numbers
- verifying each classification before validating it
- checking station names on the internet
- checking other words in Italian on the internet or somewhere else
- other actions

G02Q02

How long did you stay on SIREN that last time?

- Less than 5 minutes
- Between 5 and 15 minutes
- Between 16 and 30 minutes
- Between 31 and 59 minutes
- Between 60 and 120 minutes

- More than 120 minutes

G02Q03

Were the actions you performed that last time similar to what you usually do when on SIREN?

- Yes, it is basically what I always do.
- Yes, more or less.
- Not really
- Not at all.
- I do not remember.

G02Q04

Please, write from one to three words able to describe your feelings after that session (if English is not your native language and you find it easier to express your feelings in your native language, you can use words in that language, as long as you indicate in between brackets which language it is).

G02Q05

Can you describe the way you felt when you ended that last session on SIREN? Please, write from one to three words able to describe your feelings after that session (if English is not your native language and you find it easier to express your feelings in your native language, you can use words in that language, as long as you indicate in between brackets which language it is).

2/4 SIREN and you, more in general

G03Q06

Do you keep track of the number of pages/images that you have digitized?

- Yes
- No
- I did up to a certain point, then I lost track

G03Q07

Then, how many pages/images have you digitized?

- between 1 and 10
- between 11 and 50

- between 51 and 100
- between 101 and 200
- between 201 and 500
- between 501 and 800
- more than 800
- I do not really know

G03Q08

Do you use any specific procedure or trick for data entry (for example, "cut and paste")?

- Yes
- No

G03Q09

Which ones? Please describe, if you like, your specific procedures or tricks.

G03Q10

You use your specific procedure(s) or trick(s), in order to achieve...

- speed
- accuracy
- ease
- enjoyment
- multitasking performance
- nothing specific, it is just the force of habit
- Other: ______________

G03Q12

Do you use any specific tool for data entry (for example, two screens, or the number pad)?

- Yes
- No

G03Q13

Which ones? Please mention, if you like, your specific tool(s).

G03Q14

You use your specific tool, in order to achieve ...

- speed
- accuracy

- ease
- enjoyment
- multitasking performance
- nothing specific, it is just the force of habit
- Other: _______________

G03Q16

From where do you usually work on SIREN?

- from your home
- from someone's else home
- from work
- from a library or from a public study room
- from cafes or from other recreational spaces
- from public means of transportation (trains, busses, metro)
- Other: _______________

G03Q17

When you are working on SIREN, do you usually do other activities on the sides or in the background (for example, watching or listening to television, listening to music, radio, talking with someone)

- Yes
- No

G00Q18

When digitizing data for SIREN, are you worried about making mistakes?

- Not at all
- Usually not
- More or less
- A bit
- A lot

G03Q19

Do you find reading data difficult (for example, because the writing is small, because there is no reference to the days in the last months)?

- Not at all
- Usually not
- More or less

- A bit
- A lot

G03Q20

Is the digitizing process generally tiring for you?

- Not at all
- Usually not
- More or less
- A bit
- A lot

G03Q21

Since how long are you contributing to SIREN?

- less than a week
- between 1 and 2 weeks
- between 2 weeks and 1 month
- between 1 and 3 months
- between 3 and 6 months
- more than 6 months
- I do not know / remember

G03Q22

How often do you contribute to SIREN, on average?

- I did it once, just to try
- more than once, but very rarely, less than once a month
- once a month
- once every two weeks
- once a week
- 2 or 3 days a week
- between 4 and 5 days a week
- 6 days a week
- everyday

G00Q23

Reading and digitizing Italian gauging station names is somewhat difficult or awkward?

- Yes, because I do not know Italian
- Somewhat, because I do not know Italian

- Not really, even though I do not know Italian
- Not really, because I have a little knowledge of Italian
- Not at all, given I am proficient in Italian

G00Q24

Some pages/images contain maps or graphs. Do you pay attention to them?

- It never occurred to me or I never noticed them
- Not really, I only focus on the task of digitizing numbers
- Sometimes, but I usually focus on the task of digitizing numbers
- Yes, I tend to look at them, but without spending too much time on them
- Yes, I like to look at them and to spend time on them

G03Q26

Do you pay attention to other parts of the page/image besides the names and the numbers to digitize?

- No, never.
- Sometimes.
- Often.
- All the times.
- Other: ______________

G03Q25

Have you ever been intrigued by the name of the places where the gauging stations are?

- No, never.
- Sometimes, but just as an afterthought that did not really affected my task.
- Sometimes, and I looked for them on the internet, but after I finished my digitization session.
- Sometimes, and I looked for them on the internet, taking a break from the digitization session.
- Very often, and I looked for them on the internet, but after I finished my digitization session.
- Very often, and I looked for them on the internet, taking a break from the digitization session.
- Other:

G03Q27

Thinking about the first times you started to digitize pages/images on SIREN, how long did it take you to understand how to do things right?

- A lot.
- A bit.
- A little.

- Very little.
- No time at all. It was all very clear and easily manageable.

G03Q28

Can you tell us what you learnt, if anything, while contributing to SIREN? Please write few lines about what things you did not know before and now you know as a result of contributing to SIREN. It can regard anything, from the dynamics of rivers, to Italian villages names, to how to be more efficient while typing.

G03Q29

How did you discover SIREN?

- From Zooniverse's website and/or its newsletter
- Other internet sites or internet based social networks
- Other information sources NOT on the internet
- Word of mouth
- Other: ______________

G03Q30

Can you tell us what intrigued you when you discovered SIREN the first time? Please write few sentences about what pushed you or drawn you in contributing to SIREN.

G03Q31

How much do these terms apply to SIREN?

Please rate, on a scale from 1 to 5, where 1 means that the term does not apply and 5 means that applies a lot, the following terms.

accessible	1	2	3	4	5	No answer
boring	1	2	3	4	5	No answer
challenging	1	2	3	4	5	No answer
demanding	1	2	3	4	5	No answer
difficult	1	2	3	4	5	No answer
easy	1	2	3	4	5	No answer
engaging	1	2	3	4	5	No answer
fun	1	2	3	4	5	No answer
relaxing	1	2	3	4	5	No answer
satisfactory	1	2	3	4	5	No answer
time consuming	1	2	3	4	5	No answer
tiring	1	2	3	4	5	No answer

- understandable 1 2 3 4 5 No answer

G03Q32
How much do you know about the issues addressed by the SIREN Research Project, more in general? (for instance, do you know what the researchers working on SIREN do? do you know how the data you digitize will be used and with which aim?)

- 1 – Nothing
- 2
- 3
- 4
- 5 – A lot

G03Q33
Would you like to know more about SIREN receiving information and updates or finding them on Zooniverse?

- Yes
- No

G03Q34
Will a deeper knowledge of SIREN research project increase your engagement and your effort to contribute?

- Yes
- No
- I don't know

G03Q35
Is what you are asked to do on SIREN akin to activities that you are or you were used to perform for your job?

- Yes
- Partially
- No

G03Q36
Do you consider this aspect as one of the features that has drawn you toward SIREN?

- Yes
- No

G03Q37

Do you think or feel the kind of activities you perform on SIREN can affect positively your health, especially your cognitive abilities?

- Yes
- I guess
- Maybe
- Not relevantly
- I never really thought about it
- No

G03Q38

Did anyone suggest you to do activities similar to what you do on SIREN in order to keep your cognitive abilities up?

- Yes
- No

G03Q39

Do you think SIREN research project will be useful?

- Yes
- No

G03Q40

Can you expand on the previous answer, explaining why you think the SIREN research project will be useful?

3/4 Zooniverse, citizen science and you

G04Q41

Is SIREN the only project you contribute to on Zooniverse?

- Yes
- No

G04Q42

Can you write the names of the other projects you are contributing or you have contributed to on Zooniverse? If you do not remember the names, plese give a short description.

G04Q43

Are these other projects, you contribute to, similar, as for the task they require to perform, to SIREN?

- 1 - Totally different from SIREN
- 2 - Quite different
- 3 - In some ways
- 4 - Quite similar
- 5 - Basically the same

G04Q44

Is Zooniverse the only citizen science platform you have an account on?

- Yes
- No

G04Q45

Can you write the names of the other citizen science platforms you have an account on? If you do not remember the names, please give a short description.

G04Q47

Do you check the profile or activities of other Zooniverse's users or do you interact with them?

- Yes
- No

G04Q48

Have you ever checked the Talk page of Zooniverse or of SIREN?

- Yes
- No

G04Q49

Please rate, on a scale from 1 to 5, where 1 is "not a all relevant" and 5 is "very relevant", the following reasons for deciding to contribute to a citizen science project on Zooniverse or on another platform.

- the task is demanding
- the task is challenging
- the kind of task

- the project has been suggested by a person or institution you know and/or you trust
- the project is understandable
- the topic or the issues addressed by the project
- the community of contributors
- the group or the institution proposing it
- the aim of the project
- the possibility to learn something new
- the usefulness of the project
- the closeness of the task with what I am already able to do

4/4 Something about you

G05Q49

What is your current gender identity?

- Women
- Man
- Non-binary
- None of the above
- I prefer not to say

G01Q50

How old are you? (If you prefer not to answer, you can skip the question)

G05Q51

What is your nationality? (If you prefer not to answer, you can skip the question)

G05Q52

What is your employment status?

- Employed full-time
- Employed part-time
- Self-employed
- Unemployed
- Student
- Retired
- Homecarer

- Prefer not to say
- Other:

G05Q53

What is your job? (If you prefer not to answer, you can skip the question)

G05Q54

What kind of job have you had and what kind of job would you like to apply to? (If you prefer not to answer, you can skip the question)

G05Q55

What is your field of studies? (If you prefer not to answer, you can skip the question)

G05Q56

What was your job or what were your jobs before retiring? (If you prefer not to answer, you can skip the question)

G05Q57

What kind of job or jobs did you have before settling as homecarer? If you studied after highschool, what did you study? (If you prefer not to answer, you can skip the question)

G05Q58

Does your daily routine involve children, partners, relatives or friends?

- Yes
- No

G05Q59

Please, rate, on a scale from 1 to 5, where 1 is "none" and 5 is "very high", your interest in science. My interest in science is...

- 1 – None
- 2
- 3
- 4
- 5 - Very high

G05Q60

Please, rate, on a scale from 1 to 5, where 1 is "none" and 5 is "very high", your interest in arts and humanities. My interest in arts and humanities is...

- 1 – None
- 2
- 3
- 4
- 5 - Very high

Conclusions

G05Q61

Feel free to add last comments and/or feedback, if you like, regarding the questionnaire or your engagement in SIREN. In case you are available for an interview regarding your experience with SIREN you can write to paola.mazzoglio@polito.it
